# Optimization of the Preparation Process and Laboratory Performance of Dry Process SBS/Crumb Rubber Composite Modified Asphalt Mixtures

**DOI:** 10.3390/ma19204307

**Published:** 2026-10-13

**Authors:** Aikeranmu Aili, Zunqing Liu, Jian Sun, Renzhe Lei, Wei Wan, Haojie Ji

**Affiliations:** 1 School of Traffic & Logistics Engineering, Xinjiang Agricultural University, Urumqi 830052, China; 15026279411@163.com (A.A.); 15099172683@163.com (Z.L.);; 2 Key Laboratory of Transportation and Logistics Engineering in Xinjiang, Xinjiang Agricultural University, Urumqi 830052, China; 3 Xinjiang Transportation Investment and Construction Management Co., Ltd., Urumqi 830000, China

**Keywords:** SBS/crumb rubber composite modified masterbatch, dry process, response surface methodology, laboratory performance

## Abstract

In an effort to overcome the problems of modified asphalt mixture using the typical wet process, the dry process was used to modify 70# base asphalt with the SBS/crumb rubber composite modified masterbatch. Box–Behnken response surface methodology was used to create a regression model with the interactions of five factors, which gave the optimum preparation parameters. The results show that there are many factors that have substantial influence on the laboratory performance of asphalt mixture, and the created quadratic regression model has good fitting accuracy and predictive capability. The optimum preparation parameters were an asphalt–aggregate ratio of 4.5%, modifier dosage of 2%, dry mixing time of 65 s, wet mixing time of 107 s, and mixing temperature of 189 °C. Compared to the 70# base asphalt mixture and the wet-process asphalt mixtures, the dynamic stability of the dry-process asphalt mixtures is significantly improved by 503% and 36.6%, respectively. The TSR increased by 11.4% and 4.8%, respectively, as did the residual stability by 9.6% and 2%, respectively. Compared to the 70# base asphalt mixture, the maximum flexural strain increased by 46%, though it was slightly lower under the dry process than under the traditional wet process. The results confirm the practicality of the proposed optimization strategy for the dry process.

## 1. Introduction

Due to the continuous increase in traffic and the advancement of green and low-carbon development strategies, the development of high-performance asphalt paving materials and the recycling of waste resources have become key areas of research in road engineering [1,2]. Meanwhile, Xinjiang’s vast geographical coverage, long transportation distances, scattered construction areas, and complex environmental conditions impose more stringent requirements on the high- and low-temperature performance of asphalt pavements. Conventional wet-process preparation of SBS/crumb rubber composite modified asphalt relies primarily on high-speed shearing and high-temperature curing to achieve sufficient interaction between modifiers and asphalt binders [3]. Although this process can improve the high- and low-temperature performance of asphalt materials to some extent, it requires specialized equipment. Prolonged high-temperature storage and long-distance transportation may also lead to modifier segregation and performance degradation, making it difficult to ensure consistent engineering quality [4]. Therefore, optimizing the preparation process for dry-process SBS–rubber powder composite asphalt mixtures is significant for improving construction convenience, enhancing economic efficiency, and promoting green transportation infrastructure development [5].

Dry-process technology incorporates modifiers into asphalt mixtures in solid form during mixing, eliminating the need for storage and transportation of pre-manufactured modified asphalt. This provides potential advantages in terms of construction flexibility and transportation stability [6,7]. Unlike the wet process, the dry process introduces crumb rubber, SBS, or a composite modified masterbatch directly into the mixing chamber containing heated aggregates. This enables the modified materials to undergo particle breakage and dispersion, crumb rubber swelling, and interfacial interaction between modifiers and asphalt during mixture production [8,9,10]. This approach can effectively reduce segregation problems in pre-prepared modified asphalt during storage and transportation. In recent years, extensive studies have been conducted on dry-process rubber asphalt mixtures and composite modification technologies. Ge et al. [11] conducted a systematic review of the development of dry-process rubber asphalt mixtures, indicating that significant progress has been made in terms of material design, modification mechanisms, and performance evaluation. However, the dynamic interaction between rubber particles and asphalt and the influence of key processing parameters on performance development still require further clarification. Ma et al. [12] reported that, although dry-process technology can reduce the risk of segregation of rubber-modified asphalt during storage and improve construction flexibility, its performance is still strongly affected by the dispersion state of crumb rubber, the capacity for interfacial bonding, and the level of control over construction parameters. Wu et al. [13] reported that optimizing key dry-process parameters, including the content of crumb rubber, particle size, interaction time, and mixing temperature, promotes uniform dispersion and effective interaction with asphalt. This improves mixture compaction and enhances resistance to high-temperature deformation, low-temperature cracking, and fatigue.

Response surface methodology (RSM) is commonly used for predicting and optimizing multi-factor asphalt mixtures for the complex management of numerous processing parameters. Through constructing the second-order regression model of independent and dependent variables, RSM can evaluate the main and interaction impacts of each factor and find the ideal combination of parameters under multi-factor situations based on the model prediction [14]. Vatanparast et al. [15] reported that RSM can establish highly accurate predictive models of the relationship between processing parameters and asphalt mixture performance using fewer experimental trials. This enables the determination of optimal process combinations, demonstrating the efficiency and reliability of RSM in the multi-parameter optimization of asphalt mixtures. However, in dry-process technology, the modifier dosage, asphalt–aggregate ratio, dry/wet mixing duration, and mixing temperature jointly affect masterbatch dispersion, crumb rubber swelling, and the interaction between modifiers and asphalt at the interface [16,17]. Insufficient dry mixing can lead to poor dispersion, while excessively high treatment temperatures can accelerate rubber degradation and asphalt aging [18,19]. In addition, the existing life-cycle assessment studies show that the dry process eliminates the need for the preliminary preparation of modified asphalt and therefore reduces the stages of production, storage, and transportation, showing certain advantages in terms of energy consumption, pollutant emissions, and carbon footprint [20,21]. Secondly, although masterbatch processing increases initial preparation costs, the dry-process method eliminates the stages of prolonged high-temperature curing, high-shear colloidal milling, and long-distance transport with heat retention required for traditional wet-process modified asphalt; consequently, overall construction energy consumption is reduced by approximately 15–20%, offering greater economic advantages in terms of full-lifecycle construction and maintenance costs [22,23]. Some research has been conducted on individual performance indicators, but the multi-parameter interaction optimization of dry-process SBS–rubber powder composite masterbatch asphalt mixtures has not been sufficiently studied. Therefore, it is of great significance to optimize the preparation parameters of the dry process to achieve effective dispersion of modifiers and improvement of the comprehensive performance of mixes, so as to promote the engineering use of dry-process SBS–rubber powder composite modified asphalt mixtures in Xinjiang.

Based on this, this investigation focuses on AC-16 asphalt mixture, and the optimization of the dry process preparation method of SBS/crumb rubber composite modified asphalt masterbatch is studied. First, a preparation process was created, including aggregate preheating, dry mixing with masterbatch, wet mixing with base asphalt, adding mineral powder, constant temperature curing, and Marshall specimen compaction. The asphalt-aggregate ratio, modifier dosage, dry mixing time, wet mixing time, and mixing temperature were selected as the main factors; air voids (VV), voids in the mineral aggregate (VMA), voids filled with asphalt (VFA), Marshall stability, and flow value were used as response indicators, and a prediction model was established based on the Box–Behnken response surface method. Finally, the impacts of these components and their interactions were analyzed and the optimal process parameters were obtained by numerical optimization and Marshall testing. The study concludes with a comparative analysis of laboratory performance for three combinations, i.e., 70# base asphalt mixture, wet process SBS/crumb rubber composite modified asphalt mixture and dry process SBS/crumb rubber composite modified asphalt mixture.

## 2. Materials and Methods

### 2.1. Materials

The base asphalt used in this study was 70# base asphalt binder (Hebei Xinhai Chemical Group Co., Ltd., Cangzhou, Hebei, China), the fundamental properties of which are summarized in Table 1. The modifier used in the dry process was a commercially available solid SBS/crumb rubber composite modified asphalt masterbatch (SBS/crumb rubber masterbatch) supplied by Jiangsu Wenchang New Material Technology Co., Ltd. (Zhenjiang, Jiangsu, China). The masterbatch was a granular modifier prepared by premixing SBS with crumb rubber powder, and its basic properties are presented in Table 2. The target mixture type was an AC-16C asphalt mixture, for which andesite was used as both the coarse and fine aggregates and finely ground limestone powder as the mineral filler. The basic properties of the aggregates and filler are presented in Table 3 and Table 4.

### 2.2. Aggregate Gradation

**Figure 1:**
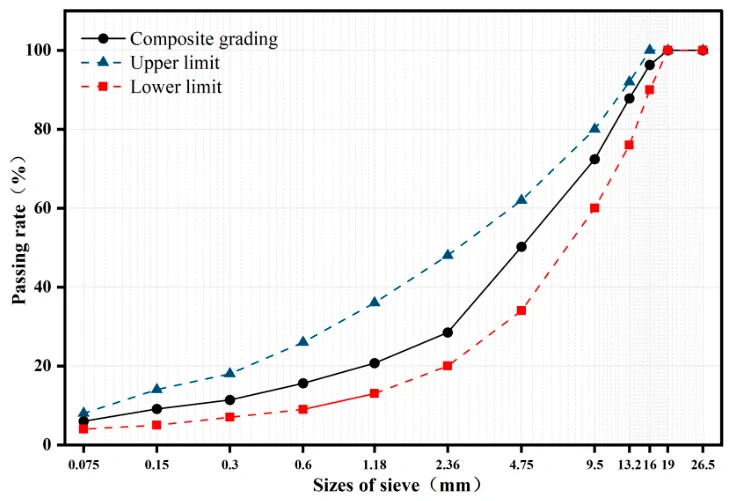
Aggregate gradation.

Figure 1 illustrates the aggregate gradation used in this study.

### 2.3. Preparation of Dry Process SBS/Crumb Rubber Composite Modified Asphalt Mixtures

**Figure 2:**
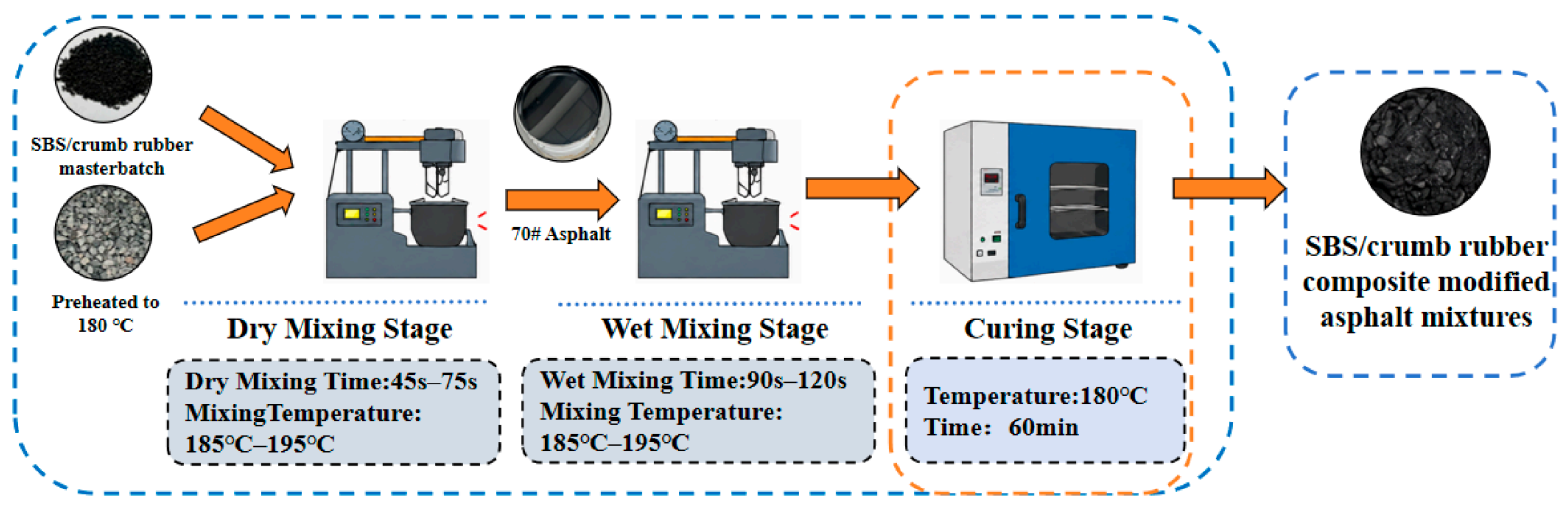
Preparation process for dry-process asphalt mixture.

The dry-process modification has two basic mixing steps. The modification principle involves preheating the aggregates to 180 °C and dry-mixing the SBS/crumb rubber masterbatch with the aggregates in a mixing vessel at 185–195 °C for 45–75 s for initial dispersion and contact with the aggregate surfaces. Then 70# base asphalt is poured for wet mixing (90–120 s), and mineral filler is added for homogeneous blending. The mixture is cured in an oven at a constant temperature of 180 °C for 60 min to obtain the SBS–rubber powder composite modified asphalt mixture. The process flow is shown in Figure 2. The curing stage is an important step of the dry process, and 180 °C for 60 min was selected mainly to encourage the swelling of the rubber powder and its full interaction with the asphalt. Long time or high temperature can aggravate the breakdown of rubber and short-term aging of asphalt, which will complicate real manufacturing, increase the production cycle, and increase project costs [11].

### 2.4. Preparation of Wet Process SBS/Crumb Rubber Composite Modified Asphalt Mixtures and 70# Asphalt Mixtures

In the preparation of wet-process modified asphalt, 15% rubber powder is added to 70# base asphalt at 160 °C and stirred; the mixture is then transferred to a high-speed shear mixer and sheared at 190 °C and 3000 rpm for 30 min. Subsequently, 3% SBS is added and sheared for 60 min, followed by the addition of 0.2% stabilizer and further shearing for 60 min (with the swelling and shearing stages occurring simultaneously). Upon completion of the swelling process, the modified asphalt is stored at 163 °C.

The wet process and 70# base asphalt mixtures used the same AC-16C aggregate grade and types of aggregates as the dry process mixture. The wet process mixture was prepared as follows: the modified asphalt was heated to 175 °C and the aggregates to 180 °C; the modified asphalt and aggregates were added to a mixing vessel at 185 °C and mixed for 90 s, then mineral filler was added and mixed for another 90 s to produce the SBS/rubber-powder composite modified asphalt mixture. Tests were carried out using this procedure to find the best asphalt–aggregate ratio, which was found to be 4.8%. In the preparation of the 70# base asphalt mixture, the asphalt was preheated to 160 °C and the aggregates to 165 °C, and the other processes were similar to those of the wet-process combination. The optimum asphalt–aggregate ratio was found to be 4.5%.

### 2.5. Response Surface Factor Design

Response surface methodology (RSM) is an optimization approach that combines experimental design and mathematical modeling. It can effectively reduce the number of experimental trials required, evaluate the interaction effects of the influencing factors, and ultimately determine the optimal conditions for the experiment [24]. To further examine the effects of dry-process parameters on volumetric properties of asphalt mixes, a Box–Behnken Design (BBD) of five factors and three levels was carried out by the Design-Expert 13 program. The five selected criteria were asphalt–aggregate ratio, modifier dosage, dry-mixing time, wet-mixing time, and mixing temperature. The asphalt–aggregate ratio was calculated based on the mass of mineral aggregate, whereas the modifier dosage was calculated based on the mass of the total mixture. In particular, the modifier dosage is the amount of SBS/crumb rubber masterbatch added into the dry process preparation, and the calculation methods are expressed in Equations (1) and (2). Table 5 presents five parameters with their level ranges. The response indicators were identified as air voids (VV), voids in mineral aggregate (VMA), voids filled with asphalt (VFA), Marshall stability (MS), and flow value.

(1) $$P_{b}=\frac{m_{b}}{m_{c}}\times 100\%$$

(2) $$P_{m}=\frac{m_{m}}{m_{a}}\times 100\%$$

where P_b_ is the asphalt-aggregate ratio; P_m_ is the modifier dosage; m_b_ is the mass of 70# asphalt; m_m_ is the mass of modifier dosage; m_c_ is the mass of mineral aggregate mass and m_a_ is the mass of the total mixture.

### 2.6. Experimental Design Scheme

A total of 43 groups of dry process SBS/crumb rubber composite modified asphalt mixtures were prepared based on the experimental design, using different combinations of processing parameters. The experimental design matrix is presented in detail in Table 6.

### 2.7. Experimental Methods

According to the Standard Test Methods of Asphalt and Asphalt Mixtures for Highway Engineering (JTG 3410–2025) [25], standard Marshall specimens were prepared for this study. The five response variables corresponding to each experimental run, as designed by the response surface methodology, were measured. The Marshall stability and flow value were then determined after conditioning the specimens in a constant-temperature water bath at 60 °C. The experimental data obtained were used to establish predictive models and determine the optimal dry process preparation parameters. The high-temperature performance, low-temperature performance, and water stability of the 70# base asphalt mixture, the wet process crumb rubber composite modified asphalt mixture, and the dry process SBS/crumb rubber composite modified asphalt mixture were then systematically evaluated for comparative analysis.

(1) High-Temperature Performance

The wheel tracking test is used to evaluate the high-temperature performance in this study. Preparation of slab specimens 300 mm × 300 mm × 50 mm shall be made in advance. The test is performed on a wheel tracking tester at a standard temperature of 60 °C ± 1 °C and a load of 780 N ± 20 N. The specimens shall be conditioned at the test temperature for at least 5 h. The test is stopped after 2520 wheel passes or when the deformation reaches 25 mm. Each specimen is tested just once at its center, and each group contains at least five specimens.

(2) Low-Temperature Performance

In the present study, low-temperature performance was evaluated by a beam bending test. Samples of the beam with dimensions of 250 mm × 40 mm × 40 mm were prepared prior to testing and conditioned for 6 h at −10 °C in an environmental chamber. Tests were then carried out on a bending testing machine with a support span of 200 mm and a loading rate of 50 mm/min. Each test group included at least four legitimate specimens.

(3) Water Stability

Freeze–thaw indirect tensile tests and immersion Marshall tests were conducted to determine moisture stability. Standard Marshall specimens were created with 50 blows on each face. For the freeze–thaw indirect tensile test, the specimens were divided into two groups; one group was held at room temperature, and the other group was vacuum saturated, then enclosed in a freezer at 18 °C for 16 h, and then immersed in a 60 °C water bath for 24 h. Finally, the groups were conditioned in a water bath at 25 °C for 2 h before indirect tensile tests at a loading rate of 50 mm/min. In the immersed splitting test, specimens were divided into two groups; one was stored at room temperature and the other was immersed in a water bath at 25 °C for 22 h. Both groups were conditioned in a water bath at 15 °C for 2 h and then subjected to indirect tensile tests at a rate of 50 mm/min.

## 3. Results and Discussion

### 3.1. Response Surface Results Analysis

#### 3.1.1. Validity Analysis

The experimental results of the 43 runs are summarized in Table 7 after data processing.

VMA characterizes the internal spatial structure of the aggregate skeleton within asphalt mixtures [26], while Marshall stability reflects the load-bearing capacity of asphalt mixtures. According to the Technical Specifications for Construction of Highway Asphalt Pavements (JTG F40–2004) [27], VMA and Marshall stability were selected as the primary response variables for detailed analysis, while the remaining three response variables were considered as constraint conditions that only needed to meet the specified requirements. As shown in Table 8 and Table 9, the coefficients of determination (R_1_^2^ and R_2_^2^) of the two models were 0.9352 and 0.9638, respectively, which is highly consistent with their corresponding adjusted coefficients of determination. This indicates that the developed models exhibited high fitting accuracy without overfitting. Furthermore, a signal-to-noise ratio greater than 4 indicates that the model can effectively describe variation trends in response values and can be used for optimization and prediction within the design space [28]. The adeq precision of the two models was 17.4012 and 21.4012, respectively, demonstrating excellent predictive capability and confirming the models’ suitability for subsequent parameter optimization.

The response surface model was fitted using a full quadratic polynomial with all main effects, quadratic terms, and interaction terms, but statistically insignificant higher-order terms were progressively eliminated in model simplification to improve model parsimony and predictive capability. In the analysis of variance (ANOVA), the F-value quantifies the size of a model term relative to random error; for a given response and degrees of freedom, a larger F-value generally implies a bigger factor effect. The P-value is a measure of the degree of agreement between the observed data and the null hypothesis. Usually, P < 0.05 is regarded as significant, P < 0.01 is considered very significant, and P > 0.05 is considered not significant [29]. The ANOVA results suggested that the quadratic regression models for VMA and Marshall stability had good goodness-of-fit with non-significant lack-of-fit terms. For the primary effects, VMA was dominated by dry mixing time (P < 0.0001, highest F-value of 24.69), while Marshall stability was heavily dependent on mixing temperature (P < 0.0001, maximum F-value of 38.39). The significant interaction effects for VMA were found to be in BD, CD, and CE. The non-significant terms were AB, AC, AD, AE, BC, BE, and DE. The non-significant terms for the Marshall stability model were AC, AE, BC, CE, DE, and E^2^, while all other terms were significant. Therefore, the quadratic regression equations of VMA and stability are obtained as illustrated in Equations (3) and (4):

Y_VMA_ = −329.403 + 11.2016A − 0.626B + 0.700C + 0.3501D + 2.938E + 0.030BD − 0.001CD − 0.003CE − 1.118A^2^ − 1.670B^2^ − 0.001C^2^ − 0.001D^2^ − 0.007E^2^ (3)

Y_MS_ = −662.008 + 47.1005A + 139.2058B − 0.4404C + 1.049D + 4.303E + 4.677AB − 0.111AD − 0.202BD − 0.541BE + 0.008CD − 2.368A^2^ − 9.205B^2^ − 0.002C^2^ − 0.006D^2^ (4)

The following is an expression of the binary quadratic regression equations for VV, VFA, and Marshall Flow:

Y_VV_ = 1606.591 − 71.190A + 17.621B − 0.350C − 2.507D − 13.772E − 1.916AB + 0.260AE + 0.042BD + 0.116BE + 0.009DE + 2.13A^2^ + 1.865B^2^ + 0.002C^2^ + 0.003D^2^ + 0.031E^2^ (5)

Y_VFA_ = −4820.650 + 232.941A − 130.7321B + 6.899C + 6.133D + 41.8299E − 12.560AB − 0.440AC + 0.228AD + 0.626BC − 0.157BD + 1.176BE + 0.010CD − 0.037CE − 0.029DE − 19.241A^2^ − 14.129B^2^ − 0.009D^2^ − 0.101E^2^ (6)

Y_FLOW_ = 199.544 − 3.610A − 19.026B + 0.137C − 0.4341D − 1.612E + 0.010AC − 0.028BC + 0.060BD + 0.083BE + 0.002DE + 0.697A^2^ + 0.001C^2^ + 0.004E^2^ (7)

#### 3.1.2. Model Evaluation

**Figure 3:**
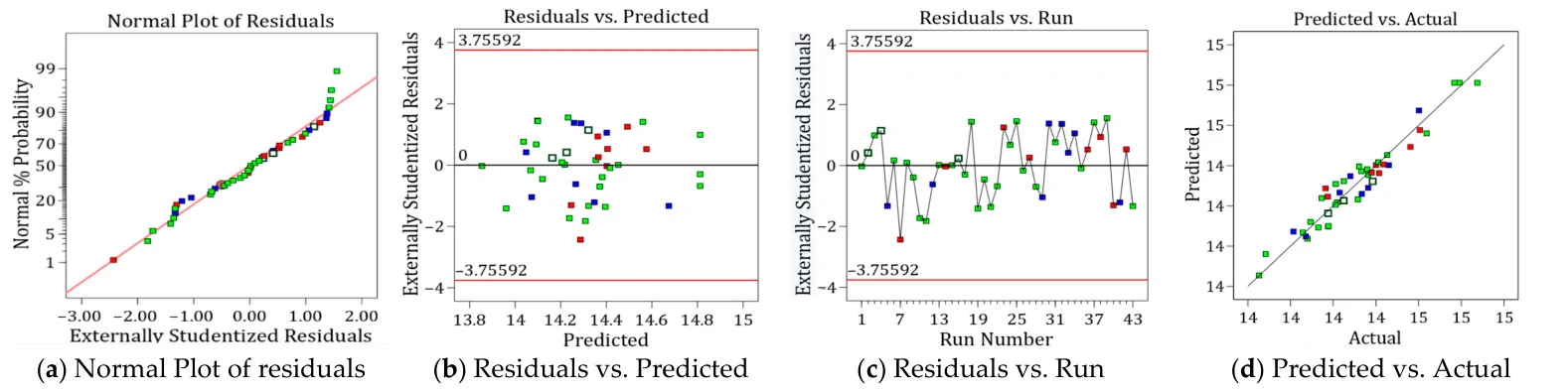
Model evaluation for VMA.

**Figure 4:**
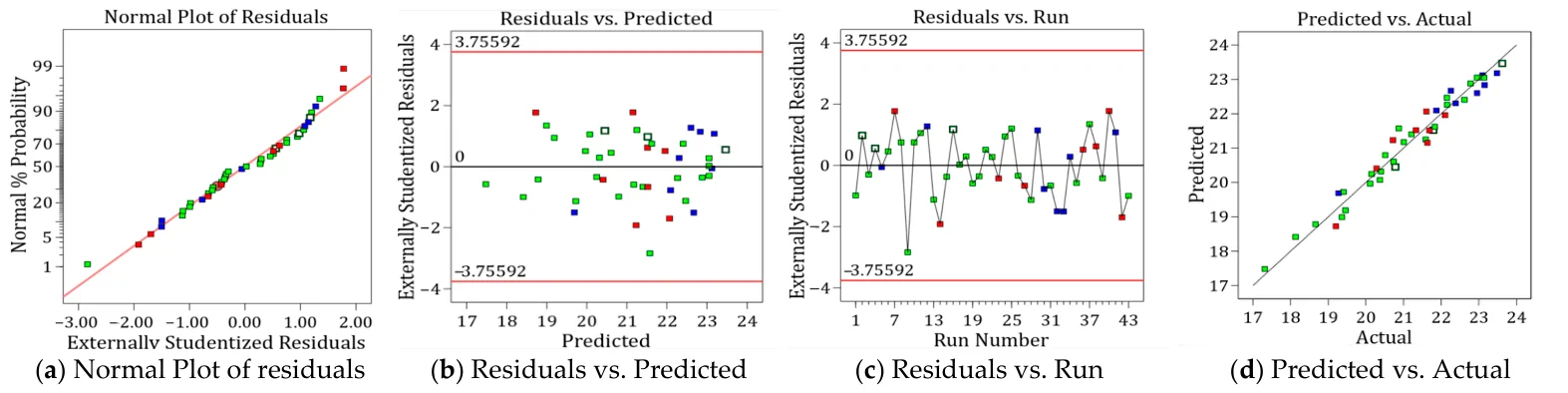
Model evaluation for Marshall stability.

Residual analysis is an essential component of diagnostics for response surface regression models. By analyzing the distribution characteristics of residuals, the fundamental assumptions of normality, independence, and homoscedasticity can be examined to evaluate the reliability of model fitting and the effectiveness of model prediction [30,31]. Residual analysis of the quadratic regression models for VMA and Marshall stability showed that the residual points closely followed the theoretical normal distribution line (see Figure 3a and Figure 4a), indicating that the normality assumption was met. The externally studentized residuals were randomly distributed within the range of ±3.76 and showed no systematic variation with respect to the experimental run order (Figure 3b,c and Figure 4b,c), confirming that the models satisfied the assumptions of homoscedasticity and independence, even in the presence of outliers. Furthermore, the scatter points between the predicted and measured values were highly concentrated around the 45° diagonal line (Figure 3d and Figure 4d), demonstrating excellent agreement and high predictive accuracy. Overall, the established response surface models exhibited satisfactory fitting performance and provided reliable support for the subsequent optimization of the preparation process.

#### 3.1.3. Interaction Effect Analysis

(1) Factor Analysis of VMA

**Figure 5:**
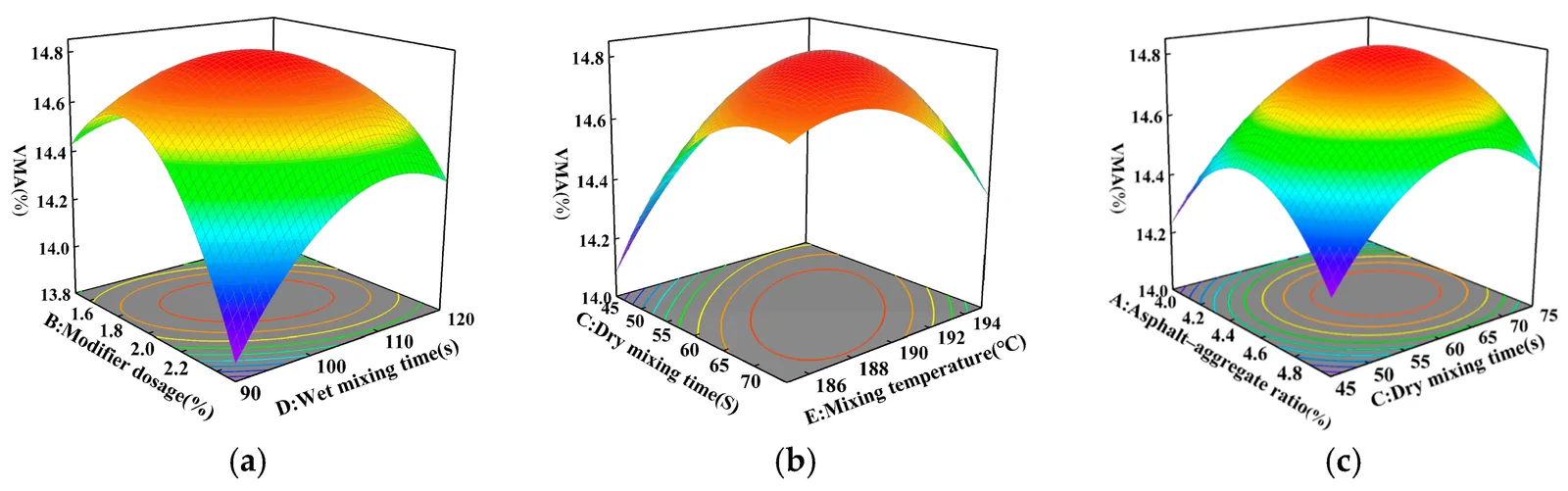
Response surface plots for VMA. (**a**) Modifier dosage vs. Wet-mixing time. (**b**) Dry-mixing time vs. Mixing temperature. (**c**) Asphalt–aggregate ratio vs. Dry-mixing time.

According to the F-values listed in Table 8, the BD and CE interaction terms had a greater effect on VMA than the other interaction terms. AC, on the other hand, had the weakest effect. As shown in Figure 5a, VMA initially increased and then decreased as the modifier dosage increased under the influence of the interaction between modifier dosage and wet mixing time (BD). A relatively high VMA value was obtained within the 1.8–2.2% modifier dosage and 100–105 s wet mixing time ranges, indicating that an appropriate combination of these two parameters contributes to the formation of a favorable aggregate skeleton structure. As illustrated in Figure 5b, VMA increased with increasing dry mixing time until approximately 68 s, after which it decreased. Meanwhile, increasing the mixing temperature from 185 to 195 °C produced a similar trend. This may be because an appropriate dry mixing duration promotes dispersion of the masterbatch on the aggregate surface, while an excessive mixing temperature increases asphalt fluidity and enhances void filling, reducing VMA. Figure 5c shows that VMA fluctuated within the range of 14.2–14.8%, with a high-value region appearing at an asphalt–aggregate ratio of 4.4–4.6% and a dry mixing time of 55–65 s. However, the corresponding F-value and P-value of this interaction term were only 0.15 and 0.69, respectively, indicating that the interaction between the asphalt–aggregate ratio and dry mixing time was insignificant. Therefore, these two factors mainly exhibited independent effects. Overall, the five factors influencing VMA were dry mixing time, modifier dosage, mixing temperature, asphalt–aggregate ratio, and wet mixing time in that order.

(2) Factor Analysis of Marshall stability

**Figure 6:**
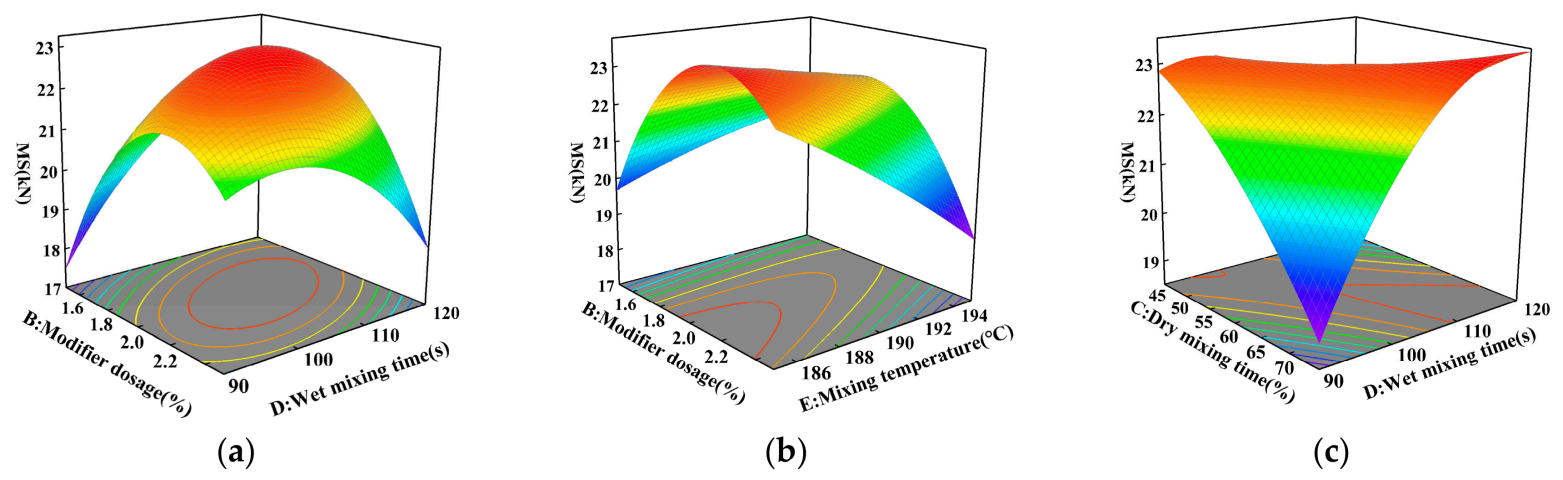
Interaction effects of factors on Marshall stability. (**a**) Modifier dosage vs. Wet-mixing time. (**b**) Modifier dosage vs. Mixing temperature. (**c**) Dry-mixing time vs. Wet-mixing time.

As shown in Figure 6a, Marshall stability initially increased, then decreased, as the modifier dosage and wet mixing time increased. The response surface exhibited a typical dome-shaped feature, and a region of high Marshall stability was observed within the 1.8–2.0% modifier dosage and 100–110 s wet mixing time ranges. Within this range, an appropriate wet-mixing duration facilitated the dispersion and interaction of the masterbatch within the asphalt mixture, thereby improving its structural stability. As illustrated in Figure 6b, Marshall stability also exhibited an initial increase followed by a decrease with variations in mixing temperature and modifier dosage, reaching a maximum value at 188–190 °C. This phenomenon may be attributed to the fact that an appropriate increase in temperature improves the interaction between the modifier, the asphalt binder, and the aggregates, thereby enhancing the stability of the mixture. However, an excessive mixing temperature may accelerate the aging of the asphalt and adversely affect the stability of the modified binder system, resulting in reduced Marshall stability. As shown in Figure 6c, Marshall stability gradually increased with increasing wet-mixing time when the dry-mixing time remained constant. This may be because the dry mixing stage primarily contributes to the initial distribution and dispersion of the modifier on aggregate surfaces. Insufficient wet mixing, however, prevents the formation of a uniform asphalt mastic–aggregate structure, ultimately reducing mixture stability. Based on the ANOVA results and response surface analysis, the order of influence of the five factors on Marshall stability was determined as follows: mixing temperature > wet mixing time > modifier dosage > asphalt–aggregate ratio > dry mixing time.

The interaction effect analysis of the remaining three response variables was conducted using the same procedure as that applied to VMA and Marshall stability. Therefore, the quadratic multiple regression models for VV, VFA, and Marshall flow were evaluated according to Table 10. The results showed that the corresponding models’ coefficients of determination (R^2^) were 0.9526, 0.9639, and 0.9688, respectively. All models exhibited statistically significant regression relationships, demonstrating satisfactory fitting performance.

The effects of the asphalt–aggregate ratio, modifier dosage, dry-mixing time, wet-mixing time, and mixing temperature on the three response variables were ranked according to significance. For VV, the order of influence of the factors was asphalt–aggregate ratio > mixing temperature > dry-mixing time > wet-mixing time > modifier dosage.

For VFA, the ranking was wet-mixing time > dry-mixing time > modifier dosage > asphalt–aggregate ratio > mixing temperature.

For Marshall flow, the order of importance was dry-mixing time > asphalt–aggregate ratio > mixing temperature > modifier dosage > wet-mixing time.

#### 3.1.4. Response Surface Optimization and Model Validation

**Figure 7:**
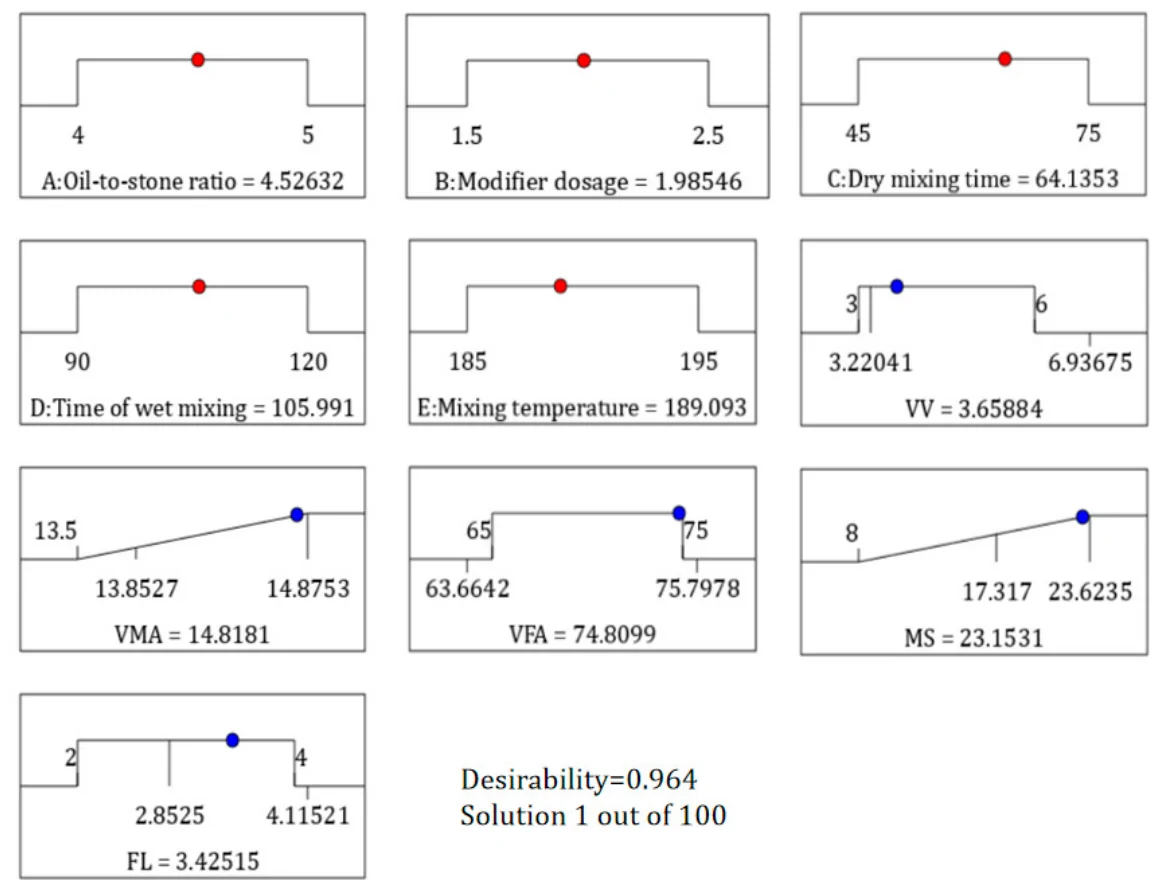
Desirability ramp for mixture design parameter optimization.

The parameters VV, VFA and flow value were set in the target ranges for the optimal mixture proportioning scheme, and VMA and MS were set for maximization according to the (JTG F40-2004) [27] (Table 11). According to the slopes of the independent variables and the response values of the prediction model (Figure 7). The software created 100 options with attractiveness values ranging from 0 to 1. The optimum mix design was achieved through systematic optimization as follows: asphalt–aggregate ratio was 4.5%, modifier dosage was 2%, dry-mixing time was 65 s, wet-mixing time was 107 s, and the mixing temperature was 189 °C, with a desirability value of 0.964. Based on this, the best scheme was designed, and the Marshall tests (average value of four prepared specimens) were carried out to verify the design. Marshall test results are shown in Table 12 and anticipated and actual values of Marshall indices are shown in Table 13.

As indicated in Table 13, the relative errors between the model-predicted values and the measured Marshall test values of the performance indicators of the dry-process SBS/rubber powder composite-modified asphalt mixture are all less than 3.46%. This proves the dependability and validity of the response surface model and validates the high accuracy of the mixing parameters and mix design proportions obtained by optimization using the response surface approach.

### 3.2. Laboratory Performance Tests of SBS/Crumb Rubber Composite Modified Asphalt Mixture

#### 3.2.1. High-Temperature Performance

**Figure 8:**
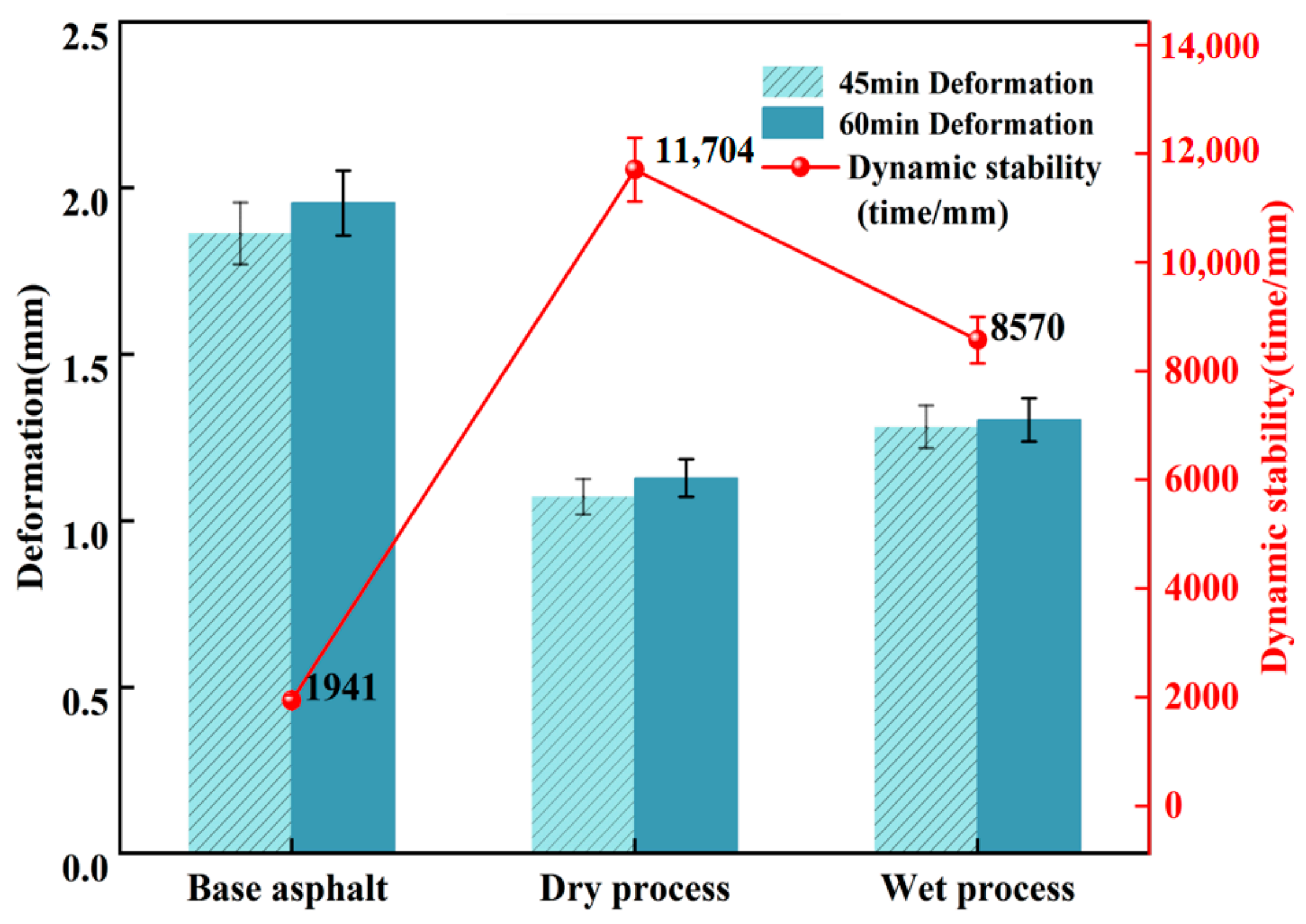
Rutting test results.

As shown in Figure 8, the dynamic stability of the dry-process SBS/crumb rubber composite modified asphalt mixtures is as high as 11,704 times/mm. This is 503% higher than that of the 70# base asphalt mixture, with deformation at 45 and 60 min reduced by 42.46% and 42.3%, respectively, compared to the dry process, indicating excellent shear flow resistance. The dynamic stability of the wet process SBS/crumb rubber composite modified asphalt mixture was 8570 times/mm, which was 36.6% lower than that of the dry process SBS/crumb rubber composite modified asphalt mixtures, and the deformation at 45 and 60 min was higher than that of the dry process mixture. Related studies have shown that the modifier is added during the dry mixing process, where it adheres to the aggregate surfaces. This improves the physical adhesion between the asphalt mastic and the aggregate particles, forming a more robust and stable skeleton-interlocking structure [32,33]. Consequently, the permanent deformation of the asphalt mixture under high-temperature and heavy-load conditions was effectively restricted at the macroscopic level.

#### 3.2.2. Low-Temperature Performance

**Figure 9:**
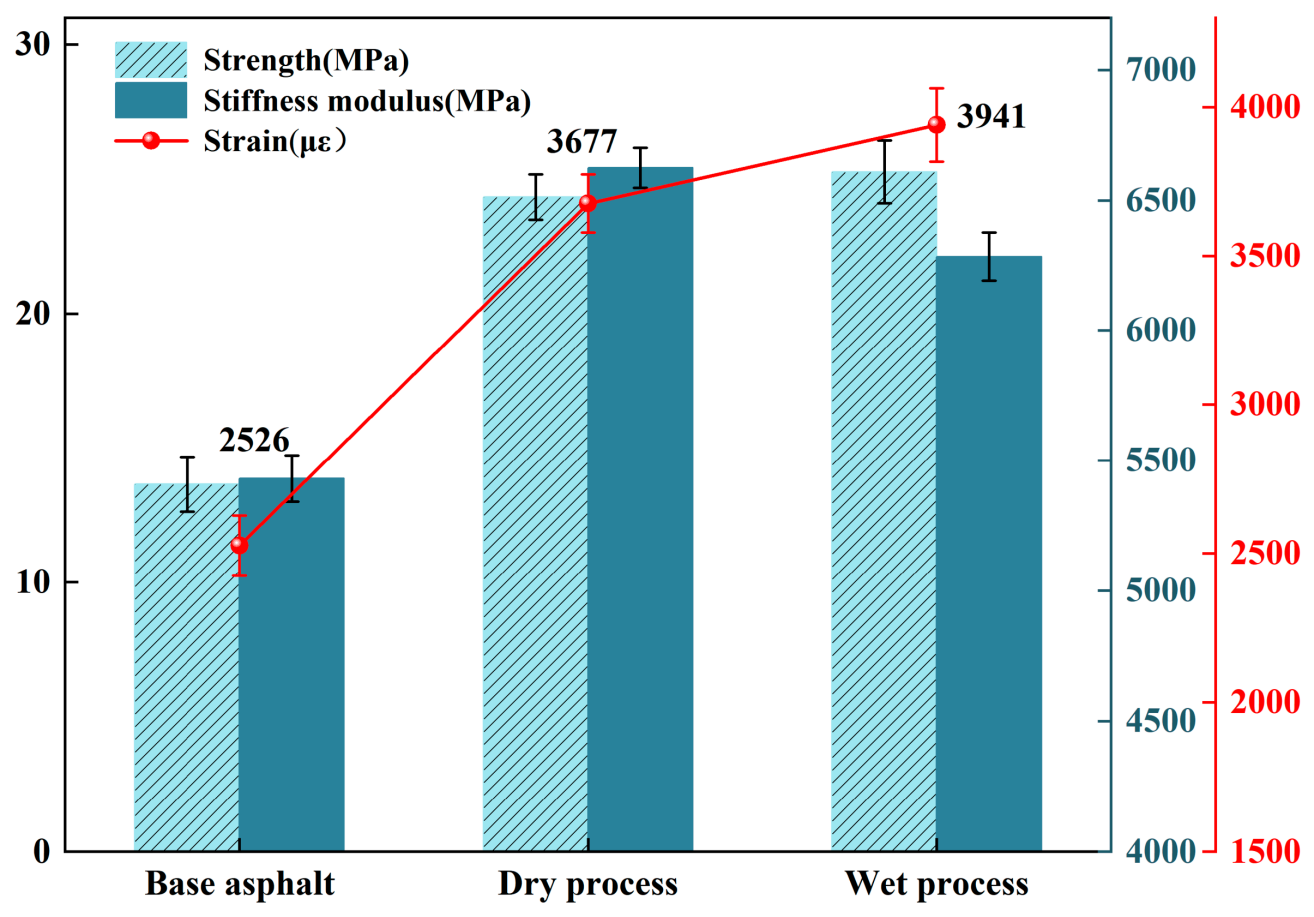
Low-temperature bending test results.

From Figure 9, it can be seen that the maximum flexural tensile strain of the dry process SBS/crumb rubber composite modified asphalt mixtures reached 3677 με. Compared with the 70# base asphalt mixture, the flexural tensile strain increased by 46%, and the flexural strength and stiffness modulus increased by 78.7% and 22%, respectively, indicating a significant improvement in low-temperature deformation capacity. However, compared with the wet process SBS/crumb rubber composite modified asphalt mixtures, the dry process SBS/crumb rubber composite modified asphalt mixtures had a 6.7% reduction in maximum flexural tensile strain, a 3.95% reduction in flexural strength, and a 5.46% increase in stiffness modulus, which means the dry-process asphalt mixture has higher low-temperature stiffness and slightly lower ultimate deformation capacity. These results suggest that the two modification systems have different deformation response characteristics. Related studies have shown that the modified asphalt prepared by the wet process generally possesses better flexibility due to the long-term, high-temperature shearing and swelling process. This results in a higher ultimate flexural tensile strain of the mixture. In contrast, dry process technology relies on synchronous mixing of the modifier with aggregates. Enhanced interlocking and adhesion at the aggregate–asphalt interface improve stress transfer and contribute to forming a stable interfacial constraint structure [34,35,36]. Although the dry-process mixture has slightly limited ultimate tensile deformation capacity, the enhanced interfacial effect provides higher structural stiffness and aggregate skeleton stability.

#### 3.2.3. Water Stability

**Figure 10:**
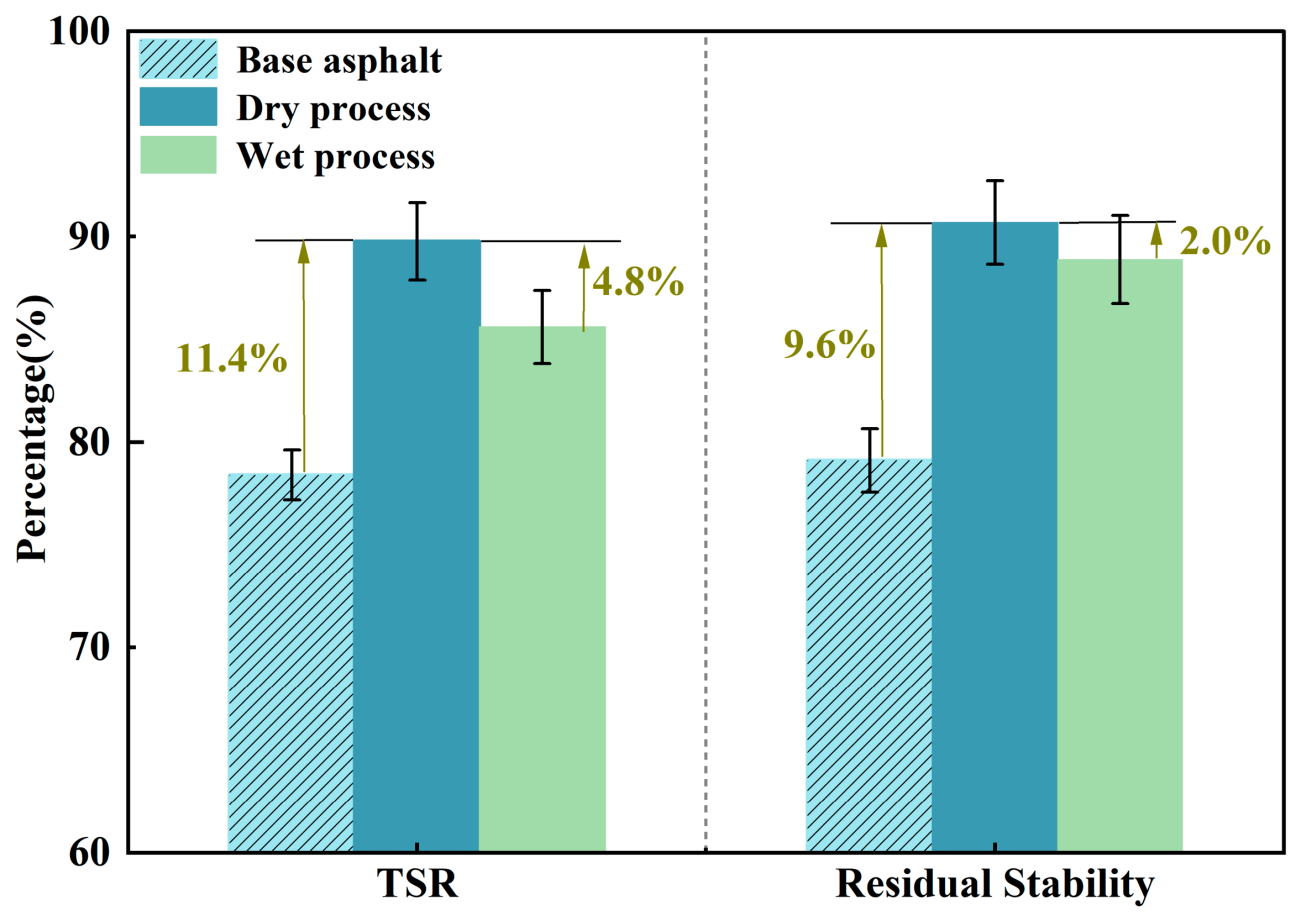
Freeze–thaw residual test results.

As Figure 10 shows, dry-process technology significantly improves the water damage resistance of the asphalt mixture. Compared with the 70# base asphalt mixture, the TSR and residual stability of the dry-process SBS/crumb rubber composite modified asphalt mixtures increased by 11.4% and 9.6%, respectively. As confirmed by statistical analysis (Table 14), the dry-process SBS/crumb rubber composite modified asphalt mixture significantly outperformed the wet-process modified asphalt mixture in both freeze–thaw splitting tensile strength ratio (increase 4.8%) and residual stability (increase 2%). According to related studies, the improvement in the performance of the mixture as a whole is due to the modifier coating the aggregate surface preferentially during dry mixing and then interacting with the asphalt during wet mixing. This enhances the interfacial bonding between the asphalt and the aggregates, creating a dense, polymer-rich barrier in the interfacial transition zone that minimizes water intrusion [37,38,39]. Consequently, the dry process mixture exhibits improved resistance to freeze–thaw damage, demonstrating the advantages of dry-process technology in terms of water stability.

## 4. Conclusions

1. The optimal dry process preparation scheme was determined, and the interaction effects of the five preparation factors were analyzed systematically. The optimal preparation parameters for the dry process SBS/crumb rubber composite modified asphalt mixtures were obtained as follows: an asphalt–aggregate ratio of 4.5%, a modifier dosage of 2%, a dry-mixing time of 65 s, a wet-mixing time of 107 s, and a mixing temperature of 189 °C.
2. The feasibility and accuracy of the response surface methodology-based optimization design were verified. Quadratic multiple regression prediction models were established for the response variables, including VV, VMA, and Marshall stability. Analysis of variance demonstrated that all models were extremely significant (*p* < 0.0001), lack-of-fit terms were not significant, and all R^2^ values were greater than 0.9, indicating satisfactory model fitting performance. Validation results showed that the relative error between predicted and Marshall experimental values was below 3.46%, confirming the reliability and feasibility of the optimal dry-process preparation parameters determined by response surface methodology.
3. The dynamic stability of the dry process SBS/crumb rubber composite modified asphalt mixtures was 503% higher than that of the 70# base asphalt mixture, and the maximum flexural tensile strain at low temperature was raised by 46%. 11.4% and 9.6% for the TSR and residual stability, respectively. The dry process SBS/crumb rubber composite modified asphalt mixture combination showed superior high-temperature stability and moisture resistance compared to the conventional wet process. Therefore, the dry process, with the benefits of process simplification and similar laboratory performance as the wet process, can directly mix the modifier into the asphalt mixture production process without independent storage and transportation of pre-produced modified asphalt, while maintaining generally similar laboratory performance as the wet process.

This study established an optimized preparation framework for dry-process SBS/crumb rubber composite modified asphalt mixtures and evaluated their high-temperature stability, resistance to low-temperature cracking, and moisture resistance. However, the long-term durability of dry process mixtures in arid climates with intense ultraviolet radiation and in saline soil environments is not well understood. Further research should therefore establish a multi-factor durability assessment framework integrating temperature variation, ultraviolet exposure, moisture, and salt-induced effects. This framework should emphasize the degradation characteristics and long-term service behavior of dry-process SBS/crumb rubber composite modified asphalt mixtures under coupled environmental conditions. Particular attention should also be given to the recycling and regeneration of mixtures after prolonged use. The microstructure and composition of reclaimed asphalt could be analyzed to clarify how SBS and rubber powder age, degrade, and regenerate during environmental exposure. These efforts would facilitate the widespread application of dry-process SBS/crumb rubber composite modified asphalt mixtures in Xinjiang and other regions with harsh climatic conditions.

## Figures and Tables

**Table 1:** Fundamental properties of the base bitumen.

Property		Requirement	Result
Penetration (25 °C, 100 g, 5 s)/(0.1 mm)		60–80	71
Softening point/°C		≥45	48
Ductility (25 °C, 10 cm/min)/cm		≥25	51
Wax content/%		≤2.2	2
Flash point/°C		≥260	281
Solubility/%		≥99.5	99.72
Residue after TFOT	Retained penetration (25 °C)/%	≥61	68
	Ductility (10 °C)/cm	≥6	10

**Table 2:** Technical properties of SBS/crumb rubber masterbatch.

Property	Result
Individual particle mass/g	≤0.1
Average particle size/mm	2.5
SBS content/%	≥45
Ash content/%	≤6
Moisture content/%	≤2
Apparent density/(g/cm^3^)	0.35–0.50

**Table 3:** Basic properties of aggregates.

Property	Coarse Aggregate			Fine Aggregate
	10–20	5–10	3–5	
Aggregate crushing value/%	14	-	-	-
Flaky and elongated particles content/%	4.1	11.3	12.1	-
Apparent specific gravity	2.815	2.832	2.855	2.848
Soundness/%	5.4	5.2	7.3	
Water absorption/%	0.33	0.67	0.75	
Sand equivalent/%	-	-	-	65
Angularity/s	-	-	-	36

**Table 4:** Basic properties of mineral fillers.

Property	Result
Apparent density/(kg/m^3^)	2.64
Moisture content/%	0.2
Hydrophilic coefficient	0.82
Plasticity index/%	3.8
Appearance	Normal

**Table 5:** Factors and levels of the experimental design.

Coded Level	A: Asphalt–Aggregate Ratio/%	B: Modifier Dosage/%	C: Dry Mixing Time/s	D: Wet Mixing Time/s	E: Mixing Temperature/°C
−1	4	1.5	45	90	185
0	4.5	2	60	105	190
1	5	2.5	75	120	195

**Table 6:** Response surface experimental design.

No	A	B	C	D	E	No	A	B	C	D	E
1	4.5	2.5	60	90	190	23	4.5	2	60	90	195
2	5	2	45	105	190	24	4	2.5	60	105	190
3	4.5	2	60	105	190	25	4	1.5	60	105	190
4	4.5	2	75	120	190	26	4.5	1.5	45	105	190
5	4.5	2	75	105	185	27	4.5	2	75	105	195
6	5	2	60	120	190	28	4.5	2	45	120	190
7	4.5	2.5	60	105	195	29	4.5	2	45	105	185
8	4	2	60	120	190	30	4.5	2	60	90	185
9	4	2	75	105	190	31	5	2.5	60	105	190
10	5	2	60	90	190	32	4.5	1.5	60	105	185
11	4	2	60	90	190	33	4.5	2.5	60	105	185
12	4	2	60	105	185	34	4.5	2	60	120	185
13	4	2	45	105	190	35	4.5	1.5	60	90	190
14	5	2	60	105	195	36	4.5	2	45	105	195
15	5	2	75	105	190	37	4.5	2	75	90	190
16	4.5	2.5	75	105	190	38	4.5	2	60	120	195
17	4.5	2	60	105	190	39	5	1.5	60	105	190
18	4.5	2.5	45	105	190	40	4.5	1.5	60	105	195
19	4.5	1.5	60	120	190	41	5	2	60	105	185
20	4.5	2	45	90	190	42	4	2	60	105	195
21	4.5	1.5	75	105	190	43	4.5	2.5	60	120	190
22	4.5	2	60	105	190						

**Table 7:** Response surface experimental results.

No	VV/%	VMA/%	VFA/%	MS/KN	Flow/mm	No	VV/%	VMA/%	VFA/%	MS/KN	Flow/mm
1	4.72	13.85	68.26	20.52	2.85	23	3.91	14.56	72.02	20.28	3.30
2	4.07	14.25	74.36	21.80	3.60	24	6.19	14.13	70.28	19.46	3.27
3	3.81	14.88	74.55	22.95	3.36	25	4.88	14.18	63.66	21.58	3.34
4	5.02	14.38	75.65	23.62	3.82	26	4.63	14.06	74.59	20.15	3.04
5	4.82	14.60	74.10	23.10	3.69	27	4.54	14.38	67.55	21.32	3.82
6	4.10	14.36	71.29	20.74	3.72	28	5.53	14.33	73.52	19.41	3.44
7	4.67	14.16	71.80	19.21	3.65	29	5.11	14.01	71.39	23.15	3.16
8	6.00	14.21	66.94	22.62	3.33	30	6.07	14.33	67.58	21.87	3.52
9	5.27	14.36	71.10	20.87	3.60	31	3.46	14.08	64.41	21.21	3.56
10	3.22	14.15	65.39	21.83	3.74	32	4.95	14.36	72.13	19.27	3.59
11	6.07	14.21	67.88	20.37	3.38	33	5.87	14.07	66.99	22.26	3.05
12	6.94	14.23	67.10	22.95	3.31	34	5.02	14.46	72.47	22.38	3.43
13	6.14	14.22	66.73	22.15	3.38	35	4.78	14.41	65.57	17.31	3.78
14	4.06	14.40	66.76	20.72	3.91	36	4.82	14.61	75.80	22.10	3.58
15	3.77	14.45	65.51	22.16	4.12	37	4.34	14.64	67.30	19.37	3.83
16	4.39	14.18	74.85	20.78	3.42	38	5.47	14.42	68.36	21.69	3.65
17	3.48	14.79	75.51	23.06	3.38	39	4.07	14.32	70.36	18.66	3.79
18	4.56	14.18	66.88	20.40	3.44	40	4.93	14.17	65.18	21.63	3.37
19	4.47	13.88	72.94	21.01	2.92	41	3.6	14.28	68.11	23.48	3.81
20	4.54	14.09	74.10	22.78	3.30	42	4.80	14.44	67.42	21.61	3.55
21	3.60	14.32	63.79	20.11	3.86	43	5.66	14.25	70.93	18.14	3.79
22	3.7	14.77	74.81	23.14	3.31						

**Table 8:** Analysis of variance for VMA.

Factor	Sum of Squares	df	Mean Square	F-Value	P-Value	Significant
Model	1.97	20	0.0984	15.87	<0.0001	Significant
A	0.0062	1	0.0062	1	0.3280	
B	0.0403	1	0.0403	6.5	0.0183	
C	0.1531	1	0.1531	24.69	<0.0001	
D	0.0002	1	0.0002	0.026	0.8734	
E	0.038	1	0.038	6.12	0.0215	
AB	0.009	1	0.009	1.46	0.2402	
AC	0.001	1	0.001	0.156	0.6967	
AD	0.012	1	0.012	1.94	0.1781	
AE	0.0018	1	0.0018	0.2893	0.5961	
BC	0.0175	1	0.0175	2.82	0.1071	
BD	0.2135	1	0.2135	34.44	<0.0001	
BE	0.0199	1	0.0199	3.2	0.0873	
CD	0.06	1	0.06	9.69	0.0051	
CE	0.1652	1	0.1652	26.64	<0.0001	
DE	0.0187	1	0.0187	3.01	0.0968	
A^2^	0.5002	1	0.5002	80.67	<0.0001	
B^2^	1.11	1	1.11	179.83	<0.0001	
C^2^	0.2896	1	0.2896	46.71	<0.0001	
D^2^	0.4186	1	0.4186	67.52	<0.0001	
E^2^	0.2002	1	0.2002	32.29	<0.0001	
Residual	0.1364	22	0.0062			
Lack of Fit	0.13	20	0.0065	2.03	0.3812	Not Significant
Std. Dev	0.0787					
C.V. %	0.5502					
R^2^	0.9352					
Adjusted R^2^	0.8763					
Predicted R^2^	0.7461					
Adeq Precision	17.4012					
PRESS	0.5344					
BIC	−46.38					
AIC_C_	−39.36					

**Table 9:** Analysis of variance for Marshall stability.

Factor	Sum of Squares	df	Mean Square	F-Value	P-Value	Significant
Model	93.80	20	4.69	29.32	<0.0001	Significant
A	0.0649	1	0.0649	0.4059	0.5307	
B	0.3086	1	0.3086	1.93	0.1787	
C	0.0223	1	0.0223	0.1395	0.7123	
D	1.73	1	1.73	10.80	0.0034	
E	6.14	1	6.14	38.39	<0.0001	
AB	5.47	1	5.47	34.19	<0.0001	
AC	0.6715	1	0.6715	4.20	0.0526	
AD	2.79	1	2.79	17.46	0.0004	
AE	0.4992	1	0.4992	3.12	0.0912	
BC	0.0437	1	0.0437	0.2729	0.6066	
BD	9.21	1	9.21	57.56	<0.0001	
BE	7.31	1	7.31	45.71	<0.0001	
CD	14.56	1	14.56	91.00	<0.0001	
CE	0.1305	1	0.1305	0.8158	0.3762	
DE	0.2005	1	0.2005	1.25	0.2750	
A^2^	2.24	1	2.24	14.02	0.0011	
B^2^	33.89	1	33.89	211.85	<0.0001	
C^2^	1.62	1	1.62	10.13	0.0043	
D^2^	10.52	1	10.52	65.75	<0.0001	
E^2^	0.229	1	0.2294	1.43	0.2438	
Residual	3.52	22	0.16			
Lack of Fit	3.50	20	0.1750	18.97	0.0512	Not Significant
Std. Dev	0.4					
C.V. %	1.88					
R^2^	0.9638					
Adjusted R^2^	0.9310					
Predicted R^2^	0.8557					
Adeq Precision	21.4012					
PRESS	14.04					
BIC	93.39					
AIC_C_	100.4					

**Table 10:** Model evaluation parameters for response variables.

Response Variable	Model	P	R^2^	Adjusted R^2^	Predicted R^2^	Lack of Fit F-Value	Adeq Precision
VV	Quadratic	<0.0001	0.9526	0.9094	0.8134	2.55	19.42
VFA	Quadratic	<0.0001	0.9639	0.9310	0.8570	4.05	19.77
FLOW	Quadratic	<0.0001	0.9688	0.9404	0.8762	4.86	27.86

**Table 11:** Optimization criteria for model evaluation parameters.

Model Evaluation Parameters	Objectives	Lower Limit	Upper Limit	Importance
VV	In range	3	6	3
VMA	Maximize	13.5	14.9	3
VFA	In range	65	75	3
MS	Maximize	8	23.6	3
FL	In range	2	4	3

**Table 12:** Marshall test results.

No	VV%	VMA%	VFA%	MS/kN	Flow/mm
1	3.71	14.46	74.33	23.79	3.76
2	3.95	14.67	73.08	23.40	3.10
3	3.75	14.49	74.11	24.17	3.42
4	3.76	14.50	74.07	23.40	3.89
Average	3.79	14.53	73.90	23.69	3.54

**Table 13:** Comparison of predicted and measured Marshall test results.

Parameter	VV%	VMA%	VFA%	MS/kN	Flow/mm
Predicted Value	3.66	14.82	74.81	23.15	3.425
Measured Value	3.79	14.53	73.90	23.69	3.54
Relative Error/%	3.46%	1.98%	1.23%	2.27%	3.25%

**Table 14:** Significance analysis of freeze–thaw splitting and residual stability tests.

Index	Mixture Group	Overall ANOVA (F-Value, P-Value)	Pairwise Comparison	P-Value	Significance
TSR	Base asphalt	F = 49.82	Dry vs. Base	0.0001	Significant
	Dry process	P < 0.0001	Wet vs. Base	0.0004	Significant
	Wet process	(R^2^ = 0.917)	Dry vs. Wet	0.0143	Significant
Residual Stability	Base asphalt	F = 73.94	Dry vs. Base	<0.0001	Significant
	Dry process	P < 0.0001	Wet vs. Base	<0.0001	Significant
	Wet process	(R^2^ = 0.943)	Dry vs. Wet	0.0159	Significant

## Data Availability

The original contributions presented in this study are included in the article. Further inquiries can be directed to the corresponding author.
